# Contemporary status of diagnostic endoluminal ultrasound and optical coherence tomography in the ureter

**DOI:** 10.1002/bco2.352

**Published:** 2024-03-19

**Authors:** Samuel Sii, Jeremy Bolton, Jake Tempo, Damien Bolton

**Affiliations:** ^1^ Department of Surgery University of Melbourne, Austin Health Melbourne Victoria Australia; ^2^ Monash University Melbourne Victoria Australia

**Keywords:** endoluminal ultrasound, optical coherence tomography, ureter

## Abstract

**Objective:**

To evaluate via a review of published literature, the efficacy of endoluminal ultrasound (ELUS) and optical coherence tomography (OCT) in the following ureteric diseases: urolithiasis, upper tract urothelial carcinoma, stricture disease and pelvic‐ureteric junction obstruction (PUJO).

**Patients and methods:**

Ureteric high‐frequency ELUS provides 360° imaging, to a depth of 20 mm, and has been demonstrated to assess ureteric stricture length, degree of fibrosis and aetiology. OCT produces high‐quality images with a penetration depth of 2 mm. ELUS has proven to be useful at the time of endopyelotomy for PUJO as it can identify crossing vessels, some not detectable on CT angiography, allowing the urologist to avoid these when making their incision. Ureteric ELUS may be utilised for submucosal ureteric stones as they are highly visible. Endoluminal ultrasound may be deployed in the case of known sub‐mucosal urolithiasis when the ureter appears stone‐free. It may help identify sub‐mucosal stones or stones within diverticulum.

**Results:**

Endoluminal ultrasound has been analysed for its use in determining muscle‐invasive urothelial carcinoma of the ureter. The PPV for ≥pT2 was only 16.7% in one study of six patients with MIBC and 76.2% in 21 patients with <pT2 disease. Analysis of OCT for staging UTUC during ureteroscopy and biopsy demonstrated sensitivity for tumour invasion of 100% and specificity of 92%, 83% of lesion staging matched with histological analysis. Imaging analysis did not match histology in three patients with large exophytic tumours that exceeded the OCT depth penetration. Due to its superficial penetration, OCT cannot reliably stage large tumours.

**Conclusions:**

Ureteric ELUS has been reported to be a useful tool in endopyelotomy, urolithiasis and stricture disease. The staging of ureteric urothelial carcinoma remains unsatisfactory with current imaging techniques and biopsy methods, and, based on the current literature, ELUS does not appear to have a strong enough PPV to determine muscle invasion. Ureteric OCT may be a useful tool in the future staging of upper tract urothelial carcinoma, particularly in differentiating the stage of small tumours. Further studies are needed in this area.

## 1 INTRODUCTION

Real‐time image acquisition of upper tract pathology acquired in the operating theatre is of great benefit to urologists. Optical coherence tomography (OCT) uses back‐scattered, near‐infrared light to penetrate tissue to a depth of 2 mm displaying cross‐sectional images on a micro‐meter scale. OCT technology is based on the principle of low‐coherence interferometry, where a low‐coherence light beam is directed on to the target tissue and the scattered back‐reflected light is combined with a reference beam. The resulting interference patterns are used to create a cross‐sectional image of the target tissue.^1^ Ultrasound (US), defined by frequencies over 20 kHz, waves can be produced by a transducer and then detected as they bounce back from tissue. US waves generated by the transducer are reflected at the surfaces of tissues of different densities, proportional to the difference in impedance. These reflected US waves are further processed to form the US image presented on the screen.^2^

Both endoluminal ultrasound (ELUS) and OCT can be deployed on intra‐luminal transducers and may provide urologists with valuable information for staging upper tract urothelial carcinoma (UTUC), assessing stricture disease, avoiding crossing vessels at endopyelotomy and locating sub‐mucosal stones. Despite ELUS being widely deployed in the diagnosis of respiratory, gastrointestinal and intravascular pathology and OCT being used increasingly in ophthalmology and cardiology, their use in urology remains low.^3^ This narrative review explores the current evidence for the use of OCT and ELUS in upper tract pathology.

## 2 LITERATURE ACQUISITION AND SELECTION

A systematic search was carried out via MEDLINE, CINAHL and Google Scholar to retrieve relevant literature from 1 January 1990 to 30 June 2023. The following medical subject headings terms and keywords were used for the search: ‘Endoluminal Ultrasound’, ‘Optical Coherence Tomography’, ‘Urology’, ‘Upper Urinary Tract’, ‘renal pelvis’ and ‘Ureter’. The review was designed to include all relevant articles detailing the use of ELUS and/or OCT in the upper urinary tract. Only articles written in English were included.

Two researchers (SS and JT) screened the titles and abstracts of articles that were identified by the search strategy to exclude irrelevant studies. They also evaluated the full text of the articles to find potentially related articles. For an article to be excluded, both reviewers had to agree that the study was not relevant. Disagreements were solved by discussion among the researchers until a consensus was reached. A total of 71 articles were initially identified and screened. Forty‐nine articles were deemed eligible and included in this review.

### 2.1 Imaging in urology

Urological imaging is centred on pyelography and computed tomography (CT) with both non‐contrast and excretory phase scans being valuable in assessing urolithiasis and filling defects respectively. Magnetic resonance imaging (MRI) is not widely used in urology beyond neoplasia. While MRI provides excellent soft tissue differentiation, with sensitivity in the staging of bladder cancer being 0.60 compared to CT 0.40, in urolithiasis MRI has inferior sensitivity (0.82) compared to CT (0.95).^4^, ^5^ Percutaneous US has limited application for ureteric wall pathology due to poor image quality consequent upon intervening pelvic organs.

### 2.2 ELUS in medicine

ELUS has developed over the past few decades to provide detailed imaging via luminal structures in gastrointestinal, respiratory, vascular and urological fields.^6^ ELUS utilises high‐frequency, catheter‐based transducers to generate ultrasonographic images of target tissue. Gastroscopic and bronchoscopic ELUS has been used for visualising and taking biopsies of suspicious tissue, and intravenous ELUS catheters are used for real‐time assessment and monitoring of atherosclerosis and thrombus, without the need for intravenous contrast.^6^ ELUS was first described in urology over 40 years ago, initially for evaluation of the prostatic urethra of and bladder cancers.^7^ ELUS has been used in the lower urinary tract to guide collagen injections and biopsies, assess intra‐sphincter and intraprostatic urethral stents and image urethral diverticulae.

**FIGURE 1:**
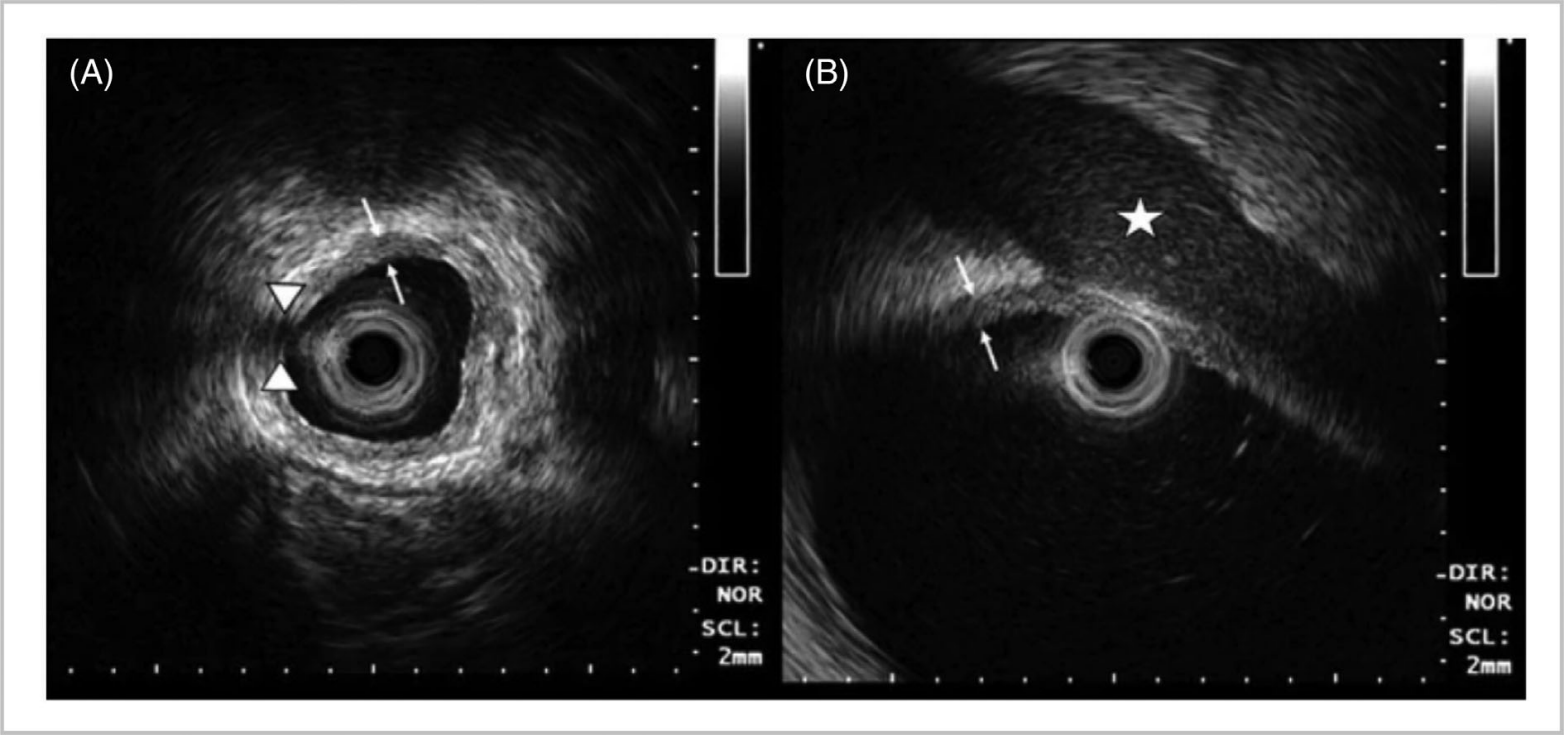
ELUS images demonstrating normal findings in varying parts of the upper urinary tract.^12^ (A) Mid ureter; (B) renal pelvis. The muscularis propria appears as a hypoechoic layer (thin arrows) surrounded by the hyperechoic fat (ureter) or renal parenchyma (renal pelvis). An acoustic shadow is seen from the guide wire (arrowheads). The renal vein is also seen (star).

ELUS also has been used in the upper urinary tract since the early 1990s.^8^ In 1994, ureteric ELUS was used in 30 consecutive cases where IV urography was inconclusive but indicated suspicion of pathology in the distal ureter.^9^ The authors reported that ELUS leads to diagnosis in 26/30 cases: 14 ureteric stones, four prostate cancers, two bladder cancers and one stricture, ureterocoele, bladder stone, detrusor hypertrophy, TURBT sequelae and one normal ureter. ELUS transducers of 9–20 MHz are fitted onto probes of between 6 and 10 Fr.^10^ High‐frequency ELUS can penetrate up to 20 mm in depth and differentiate between hypoechoic appearing muscularis and hyperechoic periureteric fat^11^ (Figure 1).

### 2.3 OCT in medicine

The use of OCT in medicine was first described in 1991.^13^ The technology has advanced rapidly in the last few decades and is now widely used in ophthalmology, cardiology and dermatology.^14^, ^15^, ^16^ Due to its ability to obtain high‐resolution images, OCT is often utilised for non‐invasive diagnosis and disease monitoring. OCT is based on the principle of low‐coherence interferometry and uses back‐scattered, near‐infrared light of wavelengths of 800–1400 nm. The interference patterns created by the two reflected light beams are used to create a cross‐sectional image of the target tissue on a micrometre scale.^1^

**FIGURE 2:**
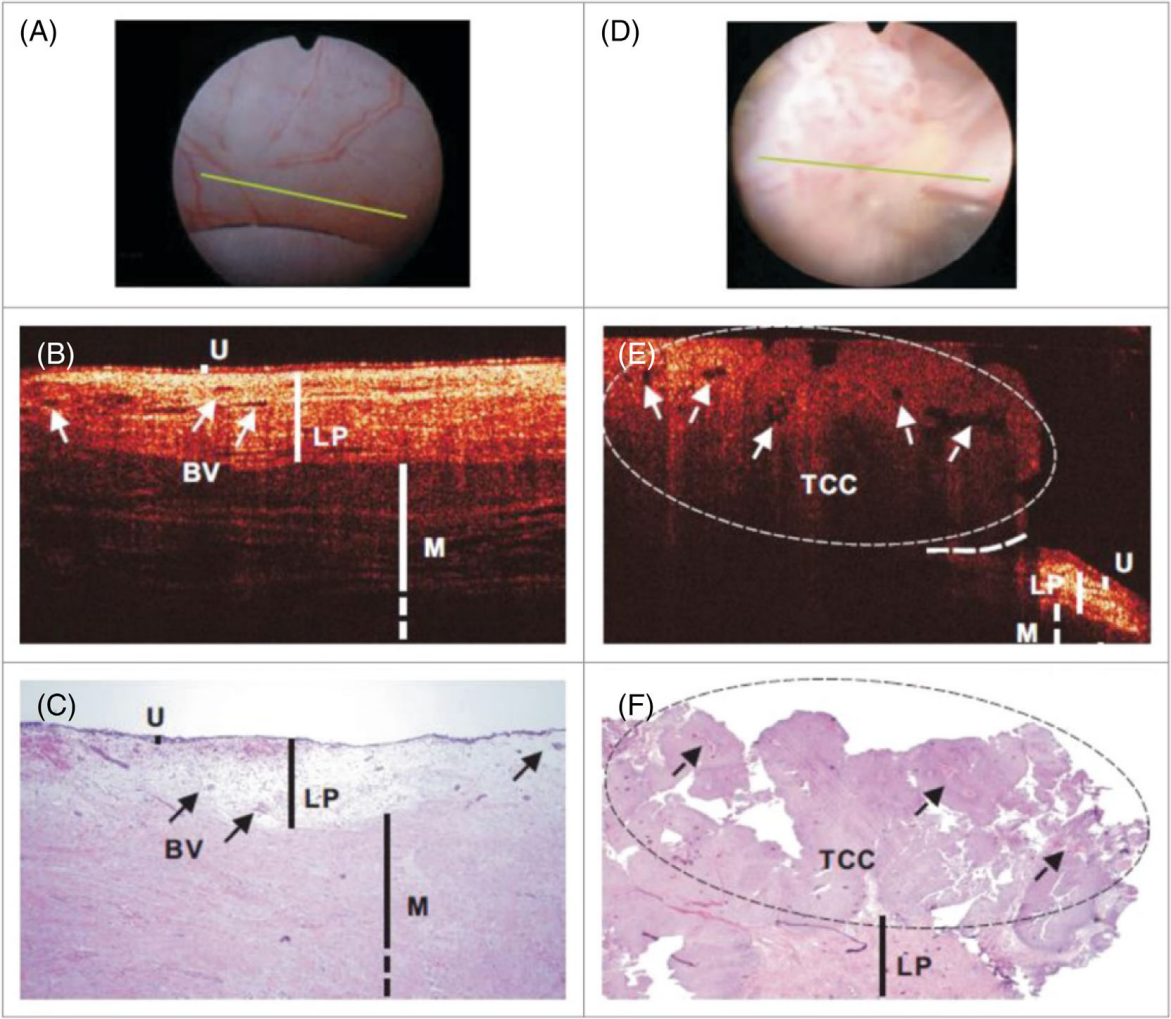
OCT images demonstrating normal urothelium compared to urothelial carcinoma.^21^ In vivo surface (A,D), cross‐sectional OCT (B,E) and H&E‐stained histologic images (C,F) of normal human bladder (A–C) versus papillary TCC (D–F). The morphologic details of normal bladder (U, urothelium; LP, lamina propria; M, upper muscularis) were clearly delineated by OCT, but those of papillary TCC diminished. Solid arrows: subsurface blood vessels; dashed arrows: papillary features; dashed circle: TCC, identified by OCT based on increased urothelial heterogeneity; dashed line: boundary with adjacent normal bladder.

In urology, OCT has been used in both the upper and lower urinary tract for evaluation of urothelium. An ex vivo 20 cm ureter can be imaged via OCT with three‐dimensional (3D) reconstruction in 90 s.^17^ An ex vivo study of OCT in porcine ureters reported 96% agreement between two independent observers differentiating between urothelium and lamina propria (*n* = 24); however, there was less than 50% agreement in differentiating between other layers of the ureteric wall.^18^, ^19^ In direct comparison between ELUS 40 MHz and OCT, chi‐square testing calculated that OCT was significantly better than ELUS distinguishing between ureteric wall layers ex vivo. There was 0.35 (kappa score) agreement between observers identifying the lamina propria and muscle layer for OCT and 0.29 (kappa score) agreement for ELUS.^18^ ELUS has not been able to reliably distinguish between urothelium and lamina propria, whereas there was 96% agreement between observers using OCT to differentiate these layers.^18^ A trial of OCT in 14 human ureters reported that the user could distinguish the ureteric wall lamina propria from the muscle layer in 98% of images; however, these findings were not compared to histology or other observers.^18^ Ex vivo comparison of five fresh human nephroureterectomy specimens with UTUC found that OCT provided better image resolution than ELUS; however, OCT was limited to a penetration depth of only 2 mm^20^ (Figure 2).

## 3 THE USE OF ELUS AND OCT IN UPPER URINARY TRACT PATHOLOGIES

In this section, we review the wide range of upper tract pathologies for which ELUS and OCT can be used.

### 3.1 UTUC

#### 3.1.1 Current trends in staging

Urothelial carcinoma is the most common malignancy of the urinary tract. Upper tract malignancies contribute 15% of cancers of the urothelium.^22^ Patients typically present with visible haematuria and undergo urine cytology examination and contrast CT with an excretory phase to examine for filling defects. On suggestion of an upper tract urothelial malignancy patients may proceed to uretero‐pyeloscopy with biopsy. Biopsy of UTUC is typically difficult due to the relatively small forceps deployable and often results in small samples and crush artefact.^23^ Often only the mucosa or lamina propria is biopsied, making staging beyond pTa/T1 rare on initial biopsy, and as a result, many urologists use tumour grade as a surrogate in determining the suitability of endoscopic, nephron‐preserving treatment strategies.^24^ Accurate staging of UTUC is important to provide patients with optimum evaluation and options in treatment. Superficial tumours may potentially be managed by laser ablation alone, while patients with T2 UTUC benefit from lymph node dissection and those with T3 disease may benefit from neoadjuvant chemotherapy prior to resection.^25^, ^26^ The sensitivity of CT with excretory phase for determining UTUC between T2 and T3 has been reported at 87.5% with a specificity of 92.9%.^27^ Overall accuracy of staging UTUC has been reported at 87.8% based on 41 cases.^28^

#### 3.1.2 Ureteric urothelial carcinoma and ELUS

Ureteric ELUS displays UTUC as a ‘well‐delineated, solid, hypoechoic area of focal thickening’ in a patient's ureter.^29^ A study of 38 patients with suspicion of UTUC on excretory phase imaging or ureteroscopy tested a method to stage upper tract malignancies using ELUS.^11^ The method of staging depended on the tumour's relation to the visible layer between hypoechoic muscularis and hyperechoic periureteric fat. Twenty‐eight patients had upper tract lesions found at uretero‐pyeloscopy ranging from 2 to 21 mm in size, 26 were UTUC, and two were fibroepithelial polyps. Two tumours, missed by ureteroscopy and RGP, were detected using ELUS. The use of ELUS for staging was successful, with five cancers seen invading the ureteric wall, three of which were confirmed histologically as pT3. Eighteen patients with suspicious masses were managed by surveillance or endoscopic treatment, and therefore, these patients could not have their full histology compared to US findings. Two false positive invasive tumours were reported due to wall thickening and oedema mimicking UTUC.

Attempts to validate ELUS for staging UTUC have been limited by small sample sizes. A study of seven patients who underwent nephroureterectomy for UTUC found that six patients were correctly staged with ELUS and one patient had non‐invasive UTUC despite ELUS suggesting invasive disease.^30^ Tumours appeared as hypoechoic masses distorting the lumen. Invasion of the ureteric muscularis was suspected when irregular, hypoechoic signals extended from the tumour into the muscularis and when there was a loss of a plane between the ureteric wall and surrounding hyperechoic tissue.^30^ The authors reported a PPV for MIUC of 66.7% and an NPV of 100% (*n* = 7). One patient was likely to be under‐staged when reported to have NMIUC on ELUS and they were excluded from the prior calculation as they received neoadjuvant chemotherapy prior to histological examination. Including this patient would bring the NPV to 80%.

The largest study to date on the use of ELUS in staging UTUC studied 53 patients under evaluation for UTUC. All ELUS was performed during the same anaesthetic following ureteroscopy.^31^ Radiologists interpreted the sonography images and were blinded to the endoscopic and histological results. Fifty‐three patients underwent ELUS without any complication, 10 patients were excluded due to receiving neoadjuvant chemotherapy, 11 did not proceed to nephroureterectomy due to metastatic disease, and five patients had uninterpretable images. Of the 27 eligible patients, ELUS correctly identified 16/21 patients with NMIUC and 1/6 with MIUC under‐staging 22.2% and over‐staging 25.9% of patients. The PPV for <pT2 was 76.2% and only 16.7% for MIUC.

#### 3.1.3 Ureteric urothelial carcinoma and OCT

Due to the ability to differentiate clearly between the layers in the ureteric wall, OCT has been trialled in the staging of UTUC.^32^ In vivo human studies report that OCT can differentiate between NMIUC and MIUC and also differentiate between low‐grade and high‐grade cancers.^33^ OCT produces images of higher axial resolution (0.01–0.015 mm) compared to 0.15 mm with a 20‐MHz ELUS transducer; however, OCT can only penetrate 2–3 mm of tissue compared to 15–20 mm with a 20‐MHz ELUS transducer.^18^ Due to the superficial penetration, OCT cannot reliably stage deep UTUC.^20^ However, given that CT pyelography has a higher sensitivity and specificity for large UTUC and is limited by missing superficial UTUC, OCT may have a role in the staging of these less invasive cancers especially given its ability to differentiate between urothelium and lamina propria.

A case series of three patients with advanced urothelial carcinoma, two located in the bladder and one in the ureter, used OCT with 3D reconstruction for assessment.^34^ For each of these carcinomas, OCT demonstrated disruption of normal structures. Analysis of OCT sensitivity and specificity for staging UTUC during the same anaesthetic as ureteroscopy and biopsy also has been conducted.^35^ Imaging was acquired on 26 ureters, all of which proceeded to nephroureterectomy or segmental ureteric resection. Sensitivity for tumour invasion was 100%, and specificity was 92%, 83% of lesion staging matched with histological analysis. Imaging analysis did not match histology in three patients with large exophytic tumours that exceeded the OCT depth penetration.

#### 3.1.4 Bladder cancer and ELUS

Small tumours of the bladder were identified by ELUS in studies initially published in the 1990s.^8^, ^36^ ELUS was used to assess sonographic staging of 32 patients with bladder UC using 5.1‐ and 7.2‐Fr probes.^37^ The hypoechoic muscularis layer was assessed to determine tumour breach (≥T2) or whether the layer was intact (<T2). Patients then underwent TURBT during the same anaesthetic, and histology was compared to ELUS findings. Under‐staging occurred in one of 18 patients categorised as <pT2 at ELUS with this patient having pT2b bladder UC histologically. Over‐staging occurred in three of 14 patients categorised as ≥T2 at ELUS with all three having pT1 disease. The four incorrectly staged tumours in this study all had broad bases >2 cm, and therefore, the US penetration was at the limit of its field.

### 3.2 Pelvic‐ureteric junction obstruction

#### 3.2.1 Imaging modalities for assessing the PUJ anatomy

Pelvic‐ureteric junction obstruction (PUJO) is a functionally significant impairment of urinary flow from the renal pelvis into the proximal ureter. ELUS has proved useful in assessing the normal pelvic‐ureteric junction (PUJ) and the occurrence and position of nearby vessels.^38^ ELUS at the PUJ located a crossing vessel in 19% of normal PUJ compared to between 53% and 79% in obstructed PUJs.^38^, ^39^, ^40^, ^41^, ^42^ Endoscopic US is suggested to be more sensitive at detecting PUJ crossing vessels than surgeons during open surgery or laparoscopic pyeloplasty.^38^ ELUS detected 15% more crossing vessels than CT imaging with IV contrast.^10^, ^43^ Another study comparing CT angiography to ELUS at detecting PUJ crossing vessels reported that ELUS is more sensitive at identifying both crossing vessels and PUJ septa.^44^ Performing ELUS of a ureter takes between 4 and 15 min.^45^

OCT may be of limited use in the detection PUJ‐adjacent vessels due to its shallow depth of penetration at 2 mm.

#### 3.2.2 The use of ELUS during endopyelotomy

ELUS can be incorporated into a catheter containing a cutting device with good preservation of image quality and therefore has proven useful in guiding the incision at endopyelotomy.^46^ Endopyelotomy has been used to relieve PUJO with success rates of 87.5% reported in 1990; however, 5‐year success rates were 61%.^39^, ^47^ The presence of vessels adjacent to the PUJ reduces the success rates of endopyelotomy and increases the risk of haemorrhage.^48^ The benefit of intra‐operative ELUS prior to endopyelotomy incision can guide urologists to change the direction of incision to avoid nearby vessels, a requirement in over half of cases.^43^

One series where endopyelotomy was treatment of choice for PUJO reported performing ELUS to assess the PUJ in 144 patients and noted that it was clinically useful in every patient.^49^ In a separate series of 37 patients, five did not proceed with incision due to sonographic findings: Two patients had unavoidable crossing vessels, one had a 3.5 cm narrowed segment, one had multiple adjacent cysts, and one had a septum with an intervening vessel.^39^ Sixteen out of 37 patients had the location of the incision changed by sonographic findings.^39^ The mean follow‐up time for this study was 10 months, and no bleeding events occurred, and there was no evidence of stricture for any patient. The success rate was reported as 87.5%.

Liu described that when ELUS was used ahead of endopyelotomy there were no cases that required blood transfusion; however, the number of cases is not stated.^10^ Hendrikx et al. reported no significant bleeding in 16 endopyelotomy cases in which ELUS was used to avoid crossing vessels.^43^ A further study used ELUS in 45 patients with PUJO for assessment ahead of endoscopic incision.^50^ An adjacent vessel was seen in 24 patients, and its location was well visualised by sonography. The sonographic images guided the choice of incision site in all patients and changed the choice of operation in five patients. The 6.2‐Fr probe used in this study failed to pass the PUJ in only one of the 46 patients. A separate case series of 36 patients with PUJO undergoing ELUS reported that endopyelotomy was not performed in 22% of patients due to unavoidable crossing vessels and the direction of incision was modified in 11%.^45^ A case report also describes the use of ELUS following PUJO recurrence after endopyelotomy.^51^ ELUS demonstrated a high insertion PUJ, which was subsequently treated with repeat endopyelotomy.

One study that compared endopyelotomy (with intraoperative ELUS) to laparoscopic pyeloplasty for the treatment of PUJO found that endopyelotomy was inferior.^52^ Laparoscopic pyeloplasty had a success rate of 94% compared to 71% undergoing endopyelotomy. Patients with PUJ crossing vessels undergoing endopyelotomy had a recurrence rate of 45%.

### 3.3 Urolithiasis

ELUS has been successful at locating calculi embedded beneath the ureteric mucosa in ureters, which appear endoscopically normal and calculi‐free^10^, ^29^; however, the clinical importance of these cases is probably low. Submucosal calculi are visible on ELUS as highly echogenic foci with acoustic shadowing, and the size, location and depth of the submucosal stone could be accurately assessed.^51^, ^53^ A study of ELUS in 20 patients referred for apparent urinary tract calcification despite ureteroscopy being negative reported that ELUS located stones in 15/20 patients.^53^

OCT is able to visualise calculi clearly as demonstrated in a study assessing the degree and location of ureteric stent encrustation in an ex vivo study.^54^ No in vivo studies of OCT's use for assessing renal tract calculi have been performed.

## 4 STRICTURE

Ureteric strictures can form as a response to trauma, urolithiasis, radiotherapy, retroperitoneal fibrosis (RPF), malignancy and hypoxia, all of which result in the deposition of collagen and fibrosis formation.^55^ The result can be obstructive kidney injury, pain and infection.^55^ Treatment may consist of dilatation, incision, excision or urinary diversion.^55^

ELUS can provide 360° imaging to calculate luminal cross‐sectional area to assess length, location, extent of fibrosis and location of adjacent vessels.^49^ ELUS or RGP can provide prognostication for strictures when assessing length, as strictures <20 mm are less likely to recur after dilatation than those >20 mm.^56^ A report of ELUS imaging of 56 patients with strictures identified useful information obtained in 49 who displayed ureteral endometriosis, RPF, surgical staples or clips.^49^ In a further study of 67 patients with ureteric stricture disease undergoing ELUS, 24 ureters showed fibrosis in the ureteric wall, displaying clearly distinct image findings from the group of 13 ureters that demonstrated ureteric wall thickening due to oedema and five ureters with narrowed segments but normal ureteric wall architecture.^57^ Seven ureters displayed normal ureteric wall architecture and compression due to extrinsic disease such as RPF. In these cases, RPF was displayed on ELUS imaging but not conventional imaging. Eight ureters were found to have both ureteric wall fibrosis and periureteric fibrosis extending into the retroperitoneum. All patients in this study had their stricture treated endoscopically with cold knife ureterotomy, electrocautery or holmium laser, and in 10 patients, the location of the cut was changed to avoid periureteric vessels detected on ELUS. In six patients, a diagnosis of ureteric endometriosis was made with the common finding of hyperechoic cystic structures filled with blood involving the ureteric wall. Also included in this study were a patient with a periureteric urinoma, two patients with iatrogenic foreign bodies in the ureteric wall, two patients with ureteric UC and 16 patients with obstructing stones not revealed on radiography prior to ELUS.

A study of the use of OCT in the urethra in 86 patients reported that adjacent vessels, degree of fibrosis and atrophy in the tissue was well visualised with OCT demonstrating that OCT can be used in luminal stricture disease. However, to date, no study on the use of OCT in upper tract stricture disease has been reported.^58^

## 5 SAFETY OF ELUS AND OPTICAL COHERENCE TOMOGRAPHY

No dedicated study has investigated the safety of ELUS or OCT; however, no serious complications were reported in any of the included studies using in vivo ELUS or OCT. In one report of 50 intra‐ureteric ELUS cases in conjuncture with ureteroscopy, one patient developed a UTI, and one patient suffered an ischaemic cardiac event.^45^

## 6 LEARNING CURVE OF ELUS AND OCT

The learning curve of interpreting ELUS and OCT images is one of the challenges of incorporating these imaging techniques into routine clinical practice. Clinicians in the field of cardiology, gastroenterology and ophthalmology are provided with structured training to interpret ELUS and OCT images throughout their training and fellowship programmes.^59^, ^60^ Furthermore, image co‐registration has been successfully used as a training tool in cardiology to reduce the learning curve of OCT and intraluminal US.^61^

Quantitative and qualitative measurements could also be clearly outlined in clinical practice guidelines as a training tool or clinical guide for clinicians interpreting images. In a cardiology training package, images along with a guide for quantitative measurements of lumen diameter, length, size, depth of plaque and calcification are provided for training purposes. Qualitative measurements such as plaque morphology, lesion morphology, thrombus and dissection are also clearly outlined.^62^ Training provided by manufacturer, courses and workshops are also essential. Cumulative Sum Control Chart (CUSUM) has been utilised to track diagnostic performance of operators in training.^63^

## 7 FUTURE DIRECTIONS FOR ELUS AND OCT

Neither ELUS nor OCT has yet become incorporated in upper urological tract pathology guidelines or routine clinical practice. Their use has been described in a variety of settings in which there is great potential; however, large studies that prove a substantial benefit mandating the use of these technologies are lacking. At present, the challenges in implementing ELUS and OCT in current practice include increased procedural time, cost and uncertainty in the relevance and practical application of endoluminal imaging results. With OCT machines costing between AUD $60 000 and $80 000 and ELUS machines costing between AUD $170 000 and $180 000, the large capital investment to obtain these technologies can be prohibitive to some.^64^, ^65^ Further prospective clinical research in this field is required to demonstrate the potential benefits of these imaging modalities and address the practical challenges posed by routine ELUS and OCT use during in the upper urinary tract.

Single‐catheter, dual‐modality systems combining intraluminal US and OCT are currently being developed and used in various medical fields.^66^ A hybrid system allows for the superior depth penetration of ELUS and superior resolution OCT to be utilised synergistically; however, fusion and co‐registration of images may be challenging due to the difficulty in identifying landmarks with OCT's limited penetration.

Novel 3D intraluminal US systems are currently being developed. These systems utilise a miniature helical ultrasonic motor to simultaneously perform rotary and linear motions, facilitating precise 3D scanning of a target structure. However, the large size of this system with the smallest transducer measuring 7 mm in diameter precludes its use in the upper urinary tract.^67^

### 7.1 Ureteric urothelial carcinoma

Published studies in the use of ELUS, OCT, CT and biopsy at staging UTUC are small and lack adequate follow‐up and control groups. Higher quality studies are difficult due to the inability to assess low‐stage UTUC due to treatment of these via in vivo destruction of the tumour and consequent lack of full thickness histological specimens. Tumours with a wide base or large volume may not be correctly staged due to inadequate ELUS penetration of tissue. Given the existing difficulty in staging UTUC, either via inadequate biopsy or CT imaging, there is potential for the use of OCT and ELUS to be explored with current generation equipment to determine whether it can better differentiate between MIUC and NMIUC. The ability to accurately stage UTUC and differentiate NMIUC and MIUC would significantly influence future practice as it allows for better selection of patients who are suitable for nephron preserving surgery.

To date, no studies have combined the use of an intra‐ureteric transducer with contrast‐enhanced US. In a study of 60 bladder tumours using an external 3D, contrast‐enhanced US to assess invasion kappa values were 0.914 for inter‐reader agreement compared to 0.717 for 3D US alone and 0.794 for contrast‐enhanced US alone.^68^ Incorporating contrast‐enhancement to ELUS may provide higher quality imaging of the ureter, increasing its sensitivity for staging UTUC.

### 7.2 PUJ

ELUS has been reported to have a higher sensitivity for detecting crossing vessels than surgery or CT imaging. Despite the higher recurrence rates in current literature, endopyelotomy still plays an important role in the management of PUJO due to the lower operative risk, shorter operative time, recovery and in‐hospital stay associated with it.^52^ The incorporation of a cutting device into the ELUS catheter allows precise endopyelotomy with real‐time imaging to avoid crossing vessels. This reduces the risk of vessel injury during endopyelotomy. To date, no studies have assessed OCTs in the assessment of the PUJ.

### 7.3 Urolithiasis

Small studies have reported that ELUS is useful for detecting ureteric sub‐mucosal stones that were not identified on plain radiographs or ureteroscopy. The use of ELUS in the ureter permits the urologist to utilise real‐time, intra‐operative data to locate and then treat impacted calculi that may not be visible at ureteroscopy.

### 7.4 Ureteric stricture disease

In descriptive studies, ELUS has performed well when used in diagnosing and prognosticating ureteric strictures. The deployment of a cutting device via the ELUS catheter allows precise treatment of stricture disease. Both ELUS and OCT may provide a urologist with additional information about a stricture's aetiology than would be available with RGP and ureteroscopy. Further prospective research is required to investigate the outcomes of ureteric stricture diseased managed endoscopically with the aid of ELUS and OCT.

## 8 CONCLUSION

The contemporary status of diagnostic ELUS and OCT in the ureter reveals promising potential for addressing challenges in urological diagnoses. ELUS, with transducers on catheters, proves valuable in various upper urinary tract pathologies, including UTUC staging, detection of bladder tumours, PUJO assessment, urolithiasis localisation, and ureteric stricture evaluation. OCT, utilising near‐infrared light, shows promise in differentiating urothelial layers, particularly in UTUC staging. While ELUS presents advantages in depth penetration, OCT excels in axial resolution. The integration of ELUS and OCT in single‐catheter, dual‐modality systems is an emerging trend.

Contrast‐enhanced US offers a potential advancement in image quality and its combination with ELUS should be also observed with interest. Despite challenges in implementation, these modalities hold potential for refining diagnostics and guiding endoscopic interventions in the upper urinary tract. However, larger, high‐quality studies are needed to establish their clinical benefits and address practical challenges in routine use.

## AUTHOR CONTRIBUTIONS

All authors contributed equally to this project.

## CONFLICT OF INTEREST STATEMENT

None.
